# Neuropsychological profile of Turner Syndrome in relation to deficits in academic and psychosocial areas. A case report

**DOI:** 10.1192/j.eurpsy.2022.1134

**Published:** 2022-09-01

**Authors:** T. Castellanos Villaverde, S. Izquierdo Pérez, A. Hospital Moreno, I. Louzao Rojas, E. Fernández-Jiménez

**Affiliations:** Hospital Universitario La Paz, Department Of Psychiatry, Clinical Psychology And Mental Health, Madrid, Spain

**Keywords:** Turner Syndrome, Executive functions, Neuropsychological Evaluation

## Abstract

**Introduction:**

Previous reviews reported an association between Turner Syndrome (TS) and a profile of deficits in some neurocognitive domains (visual-spatial domains, mathematics, and executive functions: cognitive flexibility, working memory, cognitive inhibition, and problem solving), although pointing out individual variability.

**Objectives:**

To describe the neuropsychological profile of a patient with diagnosis of TS and psychosocial difficulties attended at the Service of Psychiatry, Clinical Psychology and Mental Health at La Paz University Hospital (Madrid).

**Methods:**

A descriptive study is conducted on a single case of a 11-year-old woman with diagnosis of TS attended by a clinical psychologist at a child-adolescent Mental health center for social, family and academic difficulties. Neuropsychological assessment was completed in October, 2021. The Wechsler Intelligence Scale for Children-Five Edition (WISC-V) and Neuropsychological Assessment of Executive Functions in Children (ENFEN) batteries were administered.

**Results:**

The full-scale intelligence quotient was observed in the normal range, with lower scores in non-verbal tasks. Deficits (range from z = -2.00 to -1.75) were observed in tests of working memory, processing speed and complex problem-solving tasks. The results showed great variability in other executive functioning tasks (selective attention tasks: from z = -1.75 to -0.75; and cognitive flexibility tasks: from z = -2.25 to 0.25).

**Conclusions:**

The neurocognitive profile described in the literature was partially consistent with the results obtained in this study. The neuropsychological assessment can support the elucidation of clinical diagnostic and therapeutic factors in TS patients with relevant psychosocial or cognitive difficulties.

**Disclosure:**

No significant relationships.

